# Impact of the Use of the Beanie on the Neurodevelopmental Outcomes of Preterm Infants With Plagiocephaly: A Pilot Study

**DOI:** 10.7759/cureus.8716

**Published:** 2020-06-20

**Authors:** Noormah Mehmood, Ali Hasan, Ogochukwu Nwanne, Hajra Saeed, Ana Salazar, Christopher Berlioz, Manuel Cano, Euming Chong

**Affiliations:** 1 Pediatrics, Driscoll Children's Hospital, Corpus Christi, USA; 2 Neonatology, Driscoll Chilren's Hospital, Corpus Christi, USA

**Keywords:** developmental plagiocephaly, developmental delay, bayley scores, prematurity

## Abstract

Background

Deformational plagiocephaly (DP) is the abnormal flattening of the skull. Infants with DP have been found to have abnormal brain shape and asymmetry associated with worse neurodevelopmental outcomes on the Bayley Scales of Infant and Toddler Development-III (BSID-III) compared to those without DP. In 2009, the FDA approved a repositioning Beanie, the TortleTM (Tortle Products LLC, Greenwood Village, CO), for the prevention of flat head syndrome.

Purpose

Our goal was to assess the impact of the use of the Beanie on the neurodevelopment of preterm infants with DP admitted in the neonatal intensive care unit (NICU) using the BSID-III.

Methods

Subjects were identified using a retrospective chart review of infants during January 2013-2017. Infants of less than 32 weeks of gestational age, under 1500 g birth weight, and attending the high-risk follow-up clinic were included in the study. Neurodevelopmental assessment of patients' cognition, language, motor development using the BSID-III was performed at the 12-month and 24-month follow-up visits. The BSID-III scores for patients who used the Beanie were compared to those who did not.

Results

A total of 207 patients met the inclusion criteria. The gestational age ranged from 22.5 to 31.5 weeks with a median and mean gestational age of 26.4 weeks and 26.5 weeks respectively. Of the patients, 105 were females and 102 males. The birth weight ranged between 460 g and 1460 g with a mean of 879 g and a median of 860 g. The Beanie was used in 32 patients; 31 patients were found to use the Beanie at 12 months and 16 patients at 24 months. Of note, 12-month Bayley cognition scores were found to be statistically improved in babies who used the Beanie versus those who did not (p: 0.02). The statistical significance was not appreciated at 24 months, which could be due to a decrease in the sample size.

Conclusion

The Beanie is an inexpensive and simple way to help prevent DP in preterm infants, which in turn could improve the aforementioned outcomes.

## Introduction

Deformational plagiocephaly (DP) is the abnormal flattening of the skull caused by external factors [1,2]. This deformation can occur in utero secondary to mass effect from a twin, intrauterine masses, intrauterine factors that restrict fetal movements such as abnormal presentation, oligohydramnios, and neurological abnormalities [1]. DP also occurs in normal infants and risk factors reported include supine position during sleep, use of car seats and bouncers, lack of tummy time, and bottle propping [3-4]. With the increased incidence of sudden infantile death syndrome (SIDS) and the American Academy of Pediatrics (AAP) recommendation of the "back to sleep" campaign, there has been a reported increase in the incidence of DP.

A prospective cohort study of 200 infants that investigated the prevalence and natural history of positional plagiocephaly in normal infants reported the highest prevalence of DP at four months of age (20% of patients). The study also reported risk factors similar to previous studies, such as limited head rotation, supine sleep position, and lower activity levels [5]. The most commonly reported risk factors in a systematic review by De bock et al were: male gender, supine sleep position, limited neck rotation or preference in head position, ﬁrst-born child, lower level of activity, and lack of tummy time. In this review, the agreement between empirical studies was poor for most exposures, including supine sleep position, tummy time, and use of car seats [4].

Besides abnormal head appearance, infants with DP have also been found to have abnormal brain shape and asymmetry in a study by Collete et al.; this asymmetry and flattening of brain structures were found to be associated with issues of neurological development [1]. In a longitudinal study comparing the development of infants with and without DP at seven months and at 18 months, toddlers with DP had lower Bayley Scales of Infant Development (BSID) scores than their unaffected counterparts. In this study, motor score differences were smaller, and cognitive and language score differences were greater than those observed in infants without DP [6].

Other neurologic effects found in infants with DP from different studies include abnormal tone, visual field defects, and abnormal auditory event-related potential (ERP) [7-9]. Preterm infants generally are at higher risk of abnormal head shape compared to term infants [10]. In preterm infants, factors associated with increased risk of DP reported in one study included intracranial hemorrhage and longer duration of respiratory support. In this study, female babies were found to have increased severity of DP [10]. There have been very few studies on DP in premature babies. 

In 2009, the FDA approved a repositioning Beanie, to be used for the prevention of DP. It works by providing support to different surfaces of the head while the infant lays on that part of the head. The Beanie permits the infants with preferred head positions, to turn their heads in several positions, thus promoting the development of naturally shaped cranium and thereby preventing DP.

## Materials and methods

Patients were identified using a retrospective chart review of infants during January 2013-2017. Infants who were less than 32 weeks of gestational age, under 1500 g birth weight, and attending the high-risk follow-up clinic were included in the study. Patients with congenital cardiac defects, extracorporeal membrane oxygenation treatment (ECMO), hypoxic-ischemic encephalopathy, and severe neurologic impairment based on the Hammersmith Infant Neurological Assessment were excluded. The Beanie was used for patients diagnosed with DP based on cranial measurements by licensed physical therapists (PT). 

The Beanie was used for the management of DP only during the patients' NICU admission. Neurodevelopmental assessment of patients' cognition, language, motor development using the BSID-III was performed at the 12-month and 24-month follow-up visits at the high-risk clinic. The BSID-III scores of DP patients who used the Beanie were compared to those who did not use it. BSID-III yields composite scores reflecting the infant’s cognition, language, and motor development. The standard score was derived for each scale with a mean of 100 and an SD of 15. The BSID scores were calculated by a trained PT while in the NICU and at the high-risk clinic.

Descriptive data analysis was performed to study the characteristics of the patient population and the distribution of the data points in the data set. The mean Bayley scores for the two groups were compared by using a two-sample t-test.

## Results

A total of 207 patients met the inclusion criteria. The gestational age ranged between 22.5 and 31.5 weeks with a median and mean gestational ages of 26.4 weeks and 26.5 weeks respectively. Of the patients, 105 were females (50.7%) and 102 (49.3%) males. Patient demographics are shown in Table 1. The birth weight ranged between 460 and 1460 g with a mean of 879 g and a median of 860 g. The Beanie was used in 32 patients; 31 patients were found to use the Beanie at 12 months and 16 patients at 24 months. The composite Bayley scores for cognition, language, and motor development for patients who used the Beanie versus those who did not were compared at 12- and 24-month follow-ups at the high-risk clinic by a licensed PT. The mean composite scores for 12-month cognition showed a statistical improvement in the Bayley scores for patients who used the Beanie vs those who did not (91.3 vs 86.1). However, no statistical difference was seen in 12-month language and motor mean Bayley scores between those who used the Beanie and those who did not (87.3 and 83.6 vs 84.4 and 83.6 respectively); 24-month Bayley scores for cognition, language, and motor development for those who used the Beanie were 88.8, 79.3, and 88.3 respectively; the scores for those who did not use it were 87.8, 79.5, and 85 respectively. The mean Bayley scores at 12 and 24 months are shown in Table 2. The statistical significance was not appreciated at 24 months, which could be due to a decrease in the sample size.

**Table 1:** Patient demographics

Variables	N	Percentage of sample
Sex		
Male	102	49.3
Female	105	50.7
Race		
Caucasian	26	12.6
African American	11	5.31
Hispanic	168	81.2
Other	2	0.97
Beanie not used	164	79.2
Beanie used	31	15.0

**Table 2:** Comparison of the mean cognition, language, and motor BSID-III scores between patients who used the Beanie and the control group at 12- and 24-month follow-ups *P-value statistically significant CI: confidence interval; BSID-III: Bayley Scales of Infant and Toddler Development-III

Parameters	N	Mean Bayley score	95% CI	P-value
12-month cognition				0.02*
Beanie not used	164	86.1	83.6–88.7	
Beanie used	31	91.3	87.4–95.1	
12-month language				0.24
Beanie not used	164	84.4	82.4–86.4	
Beanie used	31	87.3	82.6–91.9	
12-month motor				0.96
Beanie not used	163	83.5	80.9–85.9	
Beanie used	31	83.6	77.7–89.4	
24-month cognition				0.76
Beanie not used	109	87.8	84.9–90.6	
Beanie used	16	88.8	82.4–95.1	
24-month language				0.90
Beanie not used	109	79.5	76.5–82.5	
Beanie used	16	79.3	72.3–86.2	
24-month motor				0.44
Beanie not used	107	85.0	82.0–88.1	
Beanie used	16	88.3	80.1–96.6	

## Discussion

DP has been associated with abnormal structural and functional neurological outcomes. Many therapies have historically been used to treat DP, including head repositioning, helmet, and even surgery in severe treatment-resistant cases [11-13]. It has been established in the published literature that patients with DP have worse Bayley scores compared to infants with normal head shapes, though the causality of this association is still under investigation [14]. The early developmental delay could lead to decreased head mobility of the infant resulting in DP, thus making DP the result of developmental delay rather than the cause.

The patients included in the study were outpatients at the high-risk clinic where their Bayley scores were calculated by a licensed PT assigned to them on each visit. Our study had comparable Bayley scores to other published data, and the Bayley scores for our study population were below the normative average of 100. Although several studies have shown an association of DP with developmental delay, no studies have been performed to show causality. The Beanie is a simple-to-use, cost-effective intervention that can improve DP by encouraging the infant to volitionally turn his or her head, thus promoting head rotation and development of the cranium and thereby treating DP. The goal of our study was to evaluate if there was an improvement in the neurodevelopmental outcomes of patients who used the Beanie for DP using the Bayley score. In this study, 207 patients were identified to have DP, and 31 used the Beanie. The Bayley scores of infants who used the Beanie were compared to those who did not use it over 24 months after discharge from the NICU. We found that the mean composite scores for 12-month cognition showed a statistical improvement in the Bayley scores for patients who used the Beanie vs those who did not. No statistical difference was appreciated in the 12-month Bayley scores for motor and language development or 24-month Bayley scores for cognition, language, and motor development. The loss of significance at 24-month follow-up could be due to a large percentage of patients being lost to follow-up, resulting in a lower sample size at 24 months.

The strengths of our study include the fact that our study population for both the control and the intervention group were comparable: all participants were born prematurely and were <1500 g in weight. We believe this allowed for a fair comparison and further strengthens the validity of the results. Additionally, the fact that the participants were studied in a longitudinal fashion for a significant period of time allowed for the measurement of the Bayley scores at 12 and 24 months. Furthermore, since we utilized the Bayley scores for measurement of neurodevelopmental outcomes, which is a well-studied tool for measurement of infant cognition, language, and motor development, we believe that there was an objective analysis of the results.

A limitation of the study was the small sample size and the large volume of patients who were lost to follow-up during the course of the study. To mitigate this weakness and to further strengthen the results and analysis of the impact of Beanie on DP and neurodevelopmental outcomes of infants, we believe that more studies should be conducted. These studies should adopt a multicenter model, with a larger sample size as well as a longer follow-up.

## Conclusions

DP is the abnormal flattening of the skull caused by external factors and has been associated with abnormal structural and poor functional neurological outcomes. In our study, the use of the Beanie showed improvement in the BSID-III scores. The Beanie can serve as an inexpensive and simple intervention to be used in the NICU and can possibly be used by a general pediatrician to help prevent and treat DP, which in turn could improve the aforementioned outcomes. The study showed significant improvement in 12-month cognition in terms of Bayley scores. This significance was not appreciated in 24-month cognition scores, which could be due to a lower sample size at 24 months. We recommend a multicenter study to further assess the effect of the Beanie on the neurological outcomes in patients with DP.
